# Isotope- and Element-Resolved Analysis of (Trans)uranium Content of Curium Targets for Heavy-Actinide Production after Intense Neutron Irradiation in the High Flux Isotope Reactor

**DOI:** 10.1021/acsmeasuresciau.6c00158

**Published:** 2026-09-25

**Authors:** Sebastian Berndt, Michael Block, Samantha K. Cary, Cristian Celis-Barros, Christoph E. Düllmann, Julie G. Ezold, Raphael Hasse, Andrea T. Loria Basto, Christoph Mokry, Thorben Niemeyer, Grace M. Power, Sebastian Raeder, Dennis Renisch, Jörg Runke, Matou Stemmler, Klaus Wendt

**Affiliations:** † Johannes Gutenberg-Universität Mainz 55099 Mainz, Germany; ‡ GSI Helmholtzzentrum für Schwerionenforschung GmbH 64291 Darmstadt, Germany; § Helmholtz-Institut Mainz 55099 Mainz, Germany; ∥ 6146 Oak Ridge National Laboratory Oak Ridge, Tennessee 37831, United States

**Keywords:** isotope production, actinides, α spectrometry, ultra-trace analysis, resonance ionization mass spectrometry

## Abstract

Curium is the heaviest element resulting from nuclear power generation and is present in spent nuclear fuel. Macroscopic amounts of transcurium elements are produced by high thermal-neutron flux irradiation of curium targets in a complex process involving many neutron captures and β^–^ decays. Competing neutron-induced fission decays enrich the resulting element mixture with lanthanides, necessitating a lanthanide-actinide separation before processing of the transcurium fraction. We report on the characterization of an aliquot of a solution from campaign 79 at the Radiochemical Engineering Development Center (REDC) at Oak Ridge National Laboratory (ORNL) taken before the chemical separation of the lanthanide fission products. The sample was characterized and quantified using α and γ spectrometry, as well as resonance ionization mass spectrometry (RIMS). The absolute content of 19 isotopes in the element range from Np to Cf was determined with typical uncertainties of 20% (1σ).

## Graphical abstract

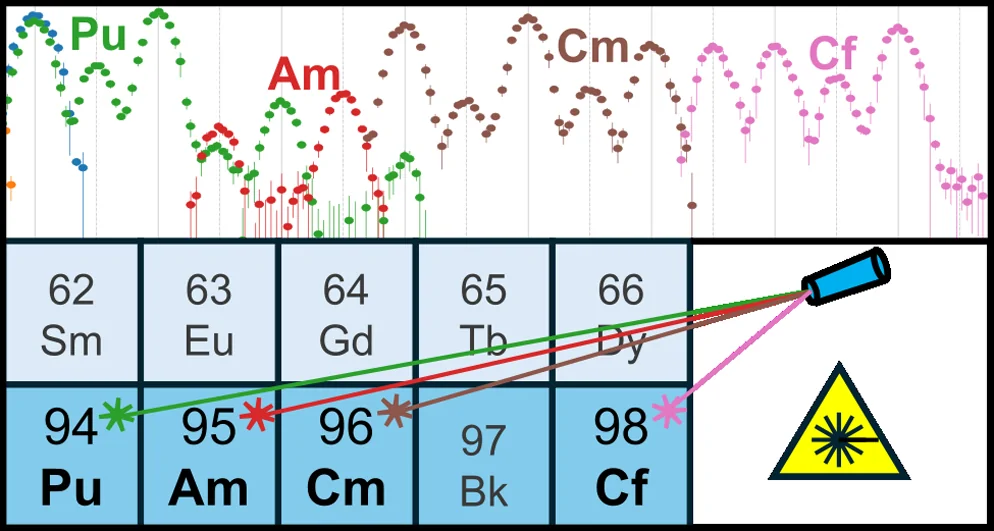

## Introduction

Actinides beyond uranium (U, Z = 92) up to Fermium (Fm, Z = 100) can be produced by neutron irradiation in nuclear reactors. The production of chemical elements above curium (Cm, Z = 96) in significant quantities requires dedicated high-flux research reactors such as the High Flux Isotope Reactor (HFIR) at Oak Ridge National Laboratory (ORNL), which features a high thermal neutron flux of up to 2.1×10^15^ n/(cm^2^ s) at a reactor power of 85 MW. HFIR produced a total amount of 10.2 g of ^252^Cf, 1.2 g of ^249^Bk, 39 mg of ^254^Es, and 15 pg of ^257^Fm between 1966 and May 2019.^1^

Transplutonium elements have a variety of uses in fundamental research and in medicine, as well as industrial applications. For example, ^252^Cf is used in many commercial applications as a neutron source owing to its spontaneous fission branch. Examples include the use as a start-up source for nuclear power reactors, or as a neutron source for neutron activation analysis (NAA) in multi-element analysis^2^ or prompt gamma neutron activation analysis (PGNAA)^3^ applied for rapid element analysis of the main components of samples such as coal, cement, or explosives.^4^ ^252^Cf as well as ^241^Am in the form of an americium–beryllium neutron source are used by the oil industry for neutron logging, which is a widespread practice for determining whether valuable reserves exist deep underground.^5^ ^252^Cf is also used in brachytherapy for the treatment of cervix tumors.^6^ In fundamental research, two new superheavy elements have been discovered in recent years by irradiating transcurium targets with a ^48^Ca ion beam. Tennessine was synthesized using ^249^Bk targets^7,8^ and named after the state of Tennessee, the home state of ORNL, thus emphasizing the critical role of actinide targets in this research field, and Oganesson was synthesized using ^249^Cf targets.^9^ In the search for the even heavier elements 119 and 120, similarly, ^249^Bk and ^249^Cf targets are used; however, instead of a ^48^Ca ion beam, the target is irradiated with a ^50^Ti ion beam.^10^ The corresponding actinides are typically required in milligram quantities in this type of research.^11^ The many applications of transcurium elements show considerable demand for these elements, and in particular for ^252^Cf. However, efficient production of these exotic elements is complex, due to the subsequent chain of many neutron capture reactions and intermittent β-decays of many involved isotopes. The main factors influencing the transmutation rates are the composition of the seed material, the neutron flux, and the duration of each irradiation cycle. Each neutron-induced reaction has a unique energy-dependent cross-section for both neutron capture and neutron-induced fission reactions. Thus, the production as well as the transmutation rates of the actinides also depend on the neutron energy spectrum of the reactor. In the case of HFIR, in addition to the above-mentioned thermal neutron flux, epithermal (1×10^14^n/(cm^2^ s)^1^) and fast (4.7×10^14^n/(cm^2^ s)^1^) neutrons are present in the irradiation region. As a consequence, some isotopes accumulate while others deplete in the target material, influencing the overall production rate of a certain isotope. Due to the complexity arising from the interplay of the large parameter set affecting the breeding process, model calculations and simulations are required for optimized irradiation planning. One approach relies on the Target Composition (TCOMP) code, which is used at ORNL to calculate and optimize the actinide yield, mainly targeting ^252^Cf.^12^

As some of the nuclear properties, which are required for the modeling are only known with limited precision, a verification and comparison of the experimentally determined yield with that expected from the simulations is desirable. Therefore, we analyzed an aliquot of the actinide-containing solution resulting from the dissolution and initial processing of irradiated Cm targets to analyze and quantify the contained actinide isotopes. In addition to classical radioanalytical methods, we used resonance ionization mass spectrometry (RIMS), an efficient and element-selective mass spectrometric method. In the following, the results of the analysis of the feed solution using RIMS as well as α and γ spectrometry performed at Johannes Gutenberg University Mainz (JGU) complement the analysis conducted at ORNL by α and γ spectrometry and inductively coupled plasma mass spectrometry (ICP-MS). These analyses provide an isotope- and element-resolved quantification of the elements neptunium, plutonium, americium, curium, and californium, including long-lived and trace isotopes.

## Experimental Setup and Sample Preparation

In campaign 79, seven mixed Cm targets containing ^244–248^Cm were irradiated at HFIR. One target was irradiated for about 102 days, five targets were irradiated for about 77 days, and the last target was irradiated for about 51 days. These irradiated targets were chemically separated at ORNL’s Radiochemical Engineering and Development Center (REDC), as described in ref 1. In preparation for the LiCl anion exchange column, some of the feed material was reprocessed through a chromatographic extraction column using DGA resin to recover and purify held-back Cm, Cf, and trivalent lanthanides. A small aliquot from the purified processed solution was taken, evaporated to dryness, and then shipped from ORNL to JGU. After arrival at the Department of Chemistry’s TRIGA site, a quantitative characterization of the feed solution was carried out with α and γ spectrometry. In both cases, the measurement lasted 16 h. For γ spectrometry, the entire sample was used, still within the shipping vial. In the case of α spectrometry, the sample was dissolved in 1 mL of 0.5 M nitric acid (HNO_3_). 1 μL (0.1%) of this sample was pipetted onto a titanium foil. For quantification using resonance ionization mass spectrometry (RIMS), three samples of the feed solution were prepared, with 1 μL (0.1%) being pipetted onto a 25-μm thick zirconium foil of 5 × 5 mm^2^ in each case. To one of these three samples, known amounts of ^249^Cf, ^248^Cm, ^241^Am, and ^240^Pu tracers were added for quantification via isotope dilution. These tracers were quantified using α spectrometry, and the added amounts are given in Table 1.

**Table 1:** ^249^Cf, ^248^Cm, ^241^Am, and ^240^Pu Amounts Added to 0.1% of the Feed Solution and Additional Isotopes Present in These Tracers

isotope	amount [×10^10^atoms]	additional isotopes
^249^Cf	4.9(5)	^245^Cm
^248^Cm	260(30)	^246^Cm,^244^Pu,^242^Pu
^241^Am	1.8(2)	^237^Np
^240^Pu	23(2)	–

The mass spectrometric studies were performed at the RISIKO mass separator^14^ at the Institute of Physics at JGU. A schematic layout of the RISIKO mass separator is shown in Figure 1. An atomic gas of the elements present on the zirconium foil is generated by resistive heating of the atomizer. This is a tantalum tube (4 mm outer diameter and 2 mm inner diameter with 35 mm length) containing the folded zirconium foil with the sample. The gaseous atoms are ionized in-source within the atomizer by an element-selective two-step laser excitation where the laser beams enter the atomizer in collinear geometry. Generated ions are then accelerated to a kinetic energy of 30 keV and analyzed according to mass-to-charge ratios in a 60° dipole magnet. A slit placed in the focal plane behind the dipole magnet limits the transmitted mass-to-charge ratio, allowing for a mass resolving power of *m*/Δ*m* ≥ 400.^14^ Mass spectrometric studies per element were performed by counting the rates of ions as a function of the magnetic field strength. Ions were detected by single ion counting using a MagneTOF detector. More details on the RISIKO mass separator are given in ref 14.

**Figure 1:**
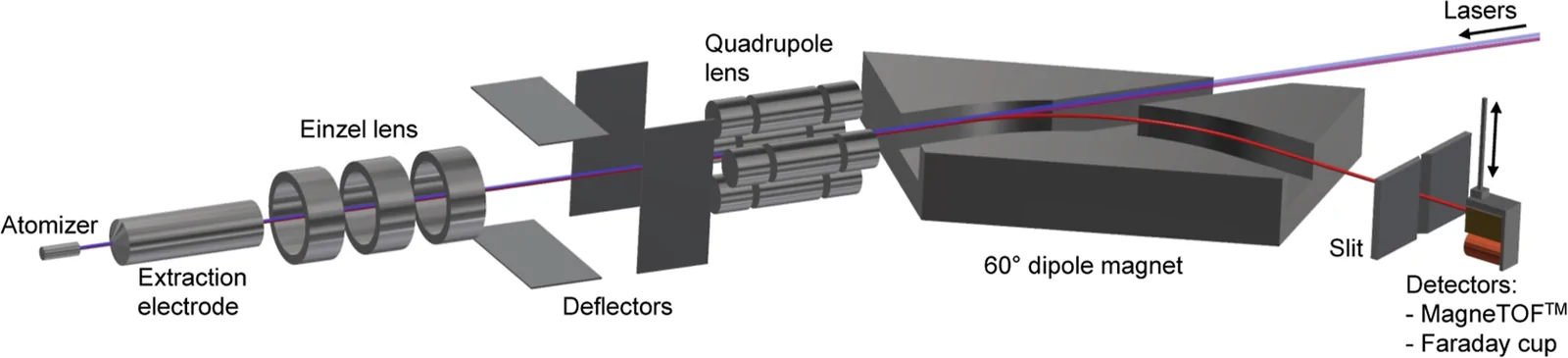
Schematic drawing of the RISIKO mass separator, adapted from ref 13. Available under a CC-BY 4.0 license via a hyperlink https://creativecommons.org/licenses/by/4.0/. Copyright N. Kneip et al. The figure depicts, from left to right, the atomizer, the extraction electrode, the ion optics, the 60° dipole magnet, and the ion detection. The ion optics consists of an einzel lens, *x*- and *y*-deflectors, and a quadrupole lens, and for the ion detection, a MagneTOF was used.

In our analysis, we additionally included below-curium elements, which may be present in the irradiated Cm targets. The laser ionization of uranium, neptunium, plutonium, americium, curium, and californium each required an individual ionization scheme. The applied wavenumbers for the first (excitation) and second (ionization) step are summarized in Table 2. Two pulsed Ti/sapphire grating lasers^15^ were used, in which second harmonic generation (SHG) was performed intracavity by laser-resonator-internal beta barium borate crystals. The average laser power was about 200 mW, the pulse duration about 30 ns, and the spectral bandwidth of these lasers was generally in the order of 5 to 7 GHz. Each Ti/sapphire laser was pumped with 17 W of 532 nm laser light produced by an individual frequency-doubled Nd/YAG laser with a 10 kHz repetition rate. Time synchronization of the laser pulses of the two lasers was achieved by a master pulse pattern generator, triggering both pump lasers.

**Table 2:** Two-Step Excitation Schemes for Uranium,^17^ Neptunium,^18^ Plutonium,^17^ Americium,^17^ Curium,^19^ and Californium;^20^ the Wavenumbers of the First Excitation Step (FES) and the Second Excitation Step (SES) are Given together with the Sublimation Enthalpies (ΔH_sub_),^21^ and the Approximate Temperatures of the Atomizer (T_A_) at which the Corresponding Actinides were Measured

elements	FES	SES	ΔH_sub_	T_A_
	[cm^–1^]	[cm^–1^]	[kJ/mol]	[°C]
U	25235.62	24754.10	533(8)	∼2100
Np	25075.15	25738.43	465.1(30)	∼2100
Pu	24188.45	24690.34	349.0(30)	∼1500
Am	23436.98	25033.38	283.8(15)	∼1200
Cm	24747.71	23590.40	384(10)	∼1600
Cf	23994.44	27669.26	196(10)	∼900

For reliable quantitative mass spectrometric studies, the optical isotope shift and the different evaporation behavior of the various actinides have to be considered. The optical isotope shift describes the shift of an optical transition between two isotopes at a given laser frequency. This shift has to be taken into account in cases where the isotope shift is wider than the bandwidth of the lasers, thus altering the ionization efficiency between different isotopes.^16^

For californium, curium, and plutonium, where long isotopic chains were measured, the wavelength of the lasers therefore had to be adjusted for each individual isotope. In contrast, for uranium, neptunium, and americium, fewer isotopes were covered, and the isotope shift is known to be comparatively small in the chosen excitation schemes used, such that a single wavelength was used to ionize all isotopes. In addition, the various actinides evaporate at different temperatures of the atomizer, with the sublimation enthalpies of the actinides (see Table 2) governing the order of the release. However, berkelium could not be measured due to the lack of a suitable two-step ionization scheme.

## Results and Discussion

At ORNL, ^238^Pu, ^244^Cm, and ^252^Cf were quantified using α spectrometry. Based on the quantification of ^252^Cf, the amount of ^249–251^Cf was determined using ICP-MS measurement of the Cf fraction.

The initial analysis of the sample at JGU was performed using γ spectroscopy of the full sample within the shipping vial. Most of the observed lines could be attributed to fission products, which are present in the sample due to the spontaneous fission of ^252^Cf. Although the 333 keV and 388 keV γ lines of ^249^Cf are clearly visible, the relative intensities of these two lines do not correspond to data reported in the literature^22^ owing to the interference of at least one of the two lines with a line of another isotope. Other actinide isotopes were not unambiguously identified. The obtained spectrum was therefore unsuitable for actinide quantification.

Five actinide isotopes were identified and quantified using α spectrometry at JGU; see Figure 2. ^252^Cf was identified via the 6118 keV and 6076 keV lines, ^250^Cf via the 6030 keV and 5989 keV lines, ^246^Cm via the 5386 keV and 5343 keV lines, ^244^Cm via the 5805 keV and 5763 keV lines, and ^238^Pu via the 5499 keV and 5456 keV lines. These isotopes were quantified by fitting the α spectrum using a multi-peak fit approach. For this purpose, an asymmetric double-sigmoidal function was used.

$$\begin{array}{ll} y= & y_{0}+\frac{A_{f}}{1+e^{-\frac{E-E_{c,f}+\omega_{1}/2}{\omega_{2}}}}\cdot \left(1-\frac{1}{1+e^{-\frac{E-E_{c,f}+\omega_{1}/2}{\omega_{3}}}}\right)\\ & +\frac{\frac{i_{s}}{i_{f}}\cdot A_{f}}{1+e^{-\frac{E-E_{c,s}+\omega_{1}/2}{\omega_{2}}}}\cdot \left(1-\frac{1}{1+e^{-\frac{E-E_{c,s}+\omega_{1}/2}{\omega_{3}}}}\right) \end{array}$$ 1

**Figure 2:**
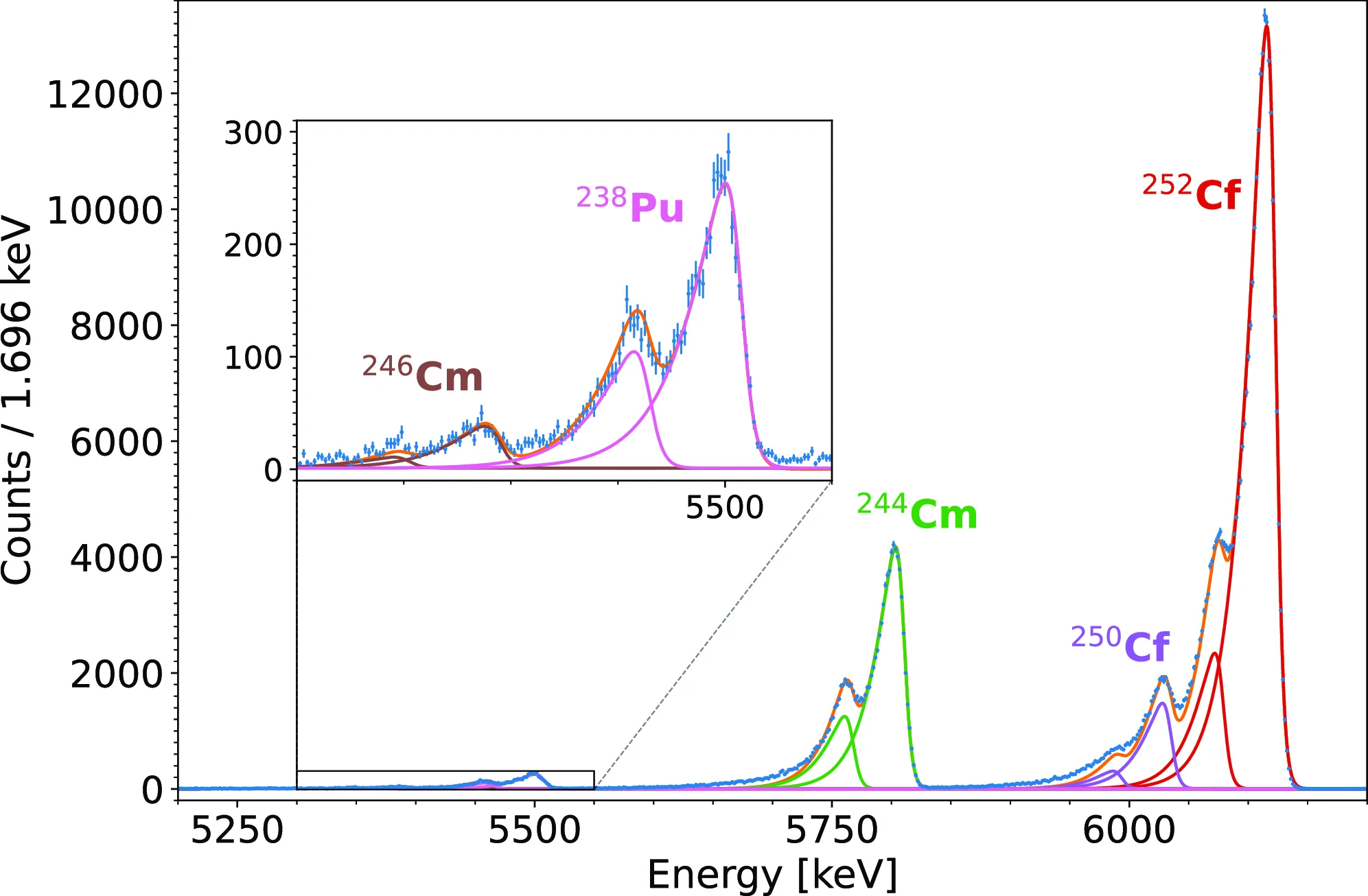
α spectrum of 0.1% of the feed solution sample measured in Mainz, fitted using a multi-peak-fit approach (orange) based on asymmetric double-sigmoidal fits for ^252^Cf (red), ^250^Cf (violet), ^246^Cm (brown), ^244^Cm (green), and ^238^Pu (pink).

This fit function is a modified version of the function described in ref 23, to include the second most prominent α line of a given isotope. Here, *y*_0_ is an offset that is 0, *E*_*c*,f_ and *E*_*c*,s_ describe the energy of the center of the two strongest α lines, and *A*_f_ is the amplitude of the strongest α line of a given isotope. For the amplitude of the second strongest α line of an isotope, the amplitude of the strongest α line was multiplied with the known ratio of the relative intensities of the two lines. ω_1_, ω_2_, and ω_3_ determine the asymmetry of the line and their values were determined by the α lines of ^244^Cm, which exhibited little interference from other α lines. The amounts of ^238^Pu, ^244,246^Cm, and ^250,252^Cf determined for the total sample are given in Table 3.

**Table 3:** Quantified Amount of ^237^Np, ^238–242,244^Pu, ^241,243^Am, ^244–248^Cm, and ^249–252^Cf via α Spectrometry and RIMS at JGU, Compared to the Quantification from ORNL; the ORNL Values and the JGU α Spectrometry Values were Decay-Corrected for Comparison to the Date of the RIMS Measurement to Account for the Decay in the Time between the Individual Measurements

isotopes	amount [atoms/mL]		
	JGU		ORNL
	α spectrometry	RIMS	
^237^Np	–	1.1(4)×10^12^	–
^238^Pu	7.0(7)×10^12^	6(1)×10^12^	8.5(8)×10^12^ ^b^
^239^Pu	–	6(1)×10^11^	–
^240^Pu	–	9(2)×10^12^	–
^241^Pu	–	1.1(2)×10^10^	–
^242^Pu	–	1.1(2)×10^11^	–
^244^Pu	–	7(2)×10^9^	–
^241^Am	–	1.2(3)×10^12^	–
^243^Am	–	7(1)×10^12^	–
^244^Cm	2.7(3)×10^13^	3.3(7)×10^13^	3.6(4)×10^13^ ^b^
^245^Cm	–	8(2)×10^11^	–
^246^Cm	5.7(6)×10^13^	5(1)×10^13^	–
^247^Cm	–	1.4(3)×10^12^	–
^248^Cm	–	1.3(3)×10^13^	–
^250^Cm	–	5(2)×10^7^	–
^249^Cf	–	6(1)×10^12^	5.9(6)×10^12^ [Table-fn t3fn3]
^250^Cf	7.0(7)×10^12^	6(1)×10^12^	6.4(6)×10^12^ [Table-fn t3fn3]
^251^Cf	–	1.3(3)×10^12^	2.2(2)×10^12^ [Table-fn t3fn3]
^252^Cf	1.2(1)×10^13^	1.4(3)×10^13^	1.5(1)×10^13^ ^b^

a Quantified using α spectrometry.

b Quantified using ICP-MS.

Using RIMS, 12 additional isotopes were identified in the sample. The measured mass spectra indicating the isotope ratios of the sample as shipped are shown in Figure 3a. This figure consists of a total of 17 individual mass scans, in which the lasers were adjusted for each element (and, when required, for individual isotopes as discussed before). The isotopes identified via RIMS are ^235,238^U, ^237^Np, ^238–242,244^Pu, ^241,243^Am, ^244–248^Cm, and ^249–252^Cf. The determined isotope ratio of ^235^U/^238^U = 9(1)×10^–3^ corresponds approximately to the ratio of natural uranium, suggesting that it originates mainly from impurities in the tantalum of the atomizer and the zirconium foil onto which the sample was pipetted. It is known from previous experiments that both materials contain traces of uranium.^24^ For the other elements, the measurement allowed for the determination of isotope ratios. For an absolute element quantification via RIMS, an isotope dilution measurement was performed by adding tracers as given in Table 1. The added tracers were characterized using RIMS and α spectrometry. This was particularly important for the ^240^Pu tracer, as ^240^Pu cannot be distinguished from ^239^Pu in the α spectrum, as the dominant α lines of these two isotopes are only 10 – 20 keV apart and could not be resolved. The RIMS measurement showed that the ^240^Pu tracer contains less than 2 orders of magnitude less ^239^Pu compared to ^240^Pu, so its contribution to the 5168 keV α line of ^240^Pu is less than 0.3% and therefore negligible. Mass spectrometric analysis of the tracers showed that, in addition to the intended isotopes, five additional isotopes were present in the tracers in detectable amounts. The ^249^Cf tracer also contained ^245^Cm, the ^248^Cm tracer contained ^246^Cm, ^244^Pu, and ^242^Pu, and the ^241^Am tracer contained ^237^Np. These are essentially the daughter nuclides of the tracer isotopes, except for ^246^Cm and its daughter, ^242^Pu. For absolute quantification, the absolute amount of one isotope of each element, i.e., ^238^Pu, ^243^Am, ^244^Cm, and ^250^Cf, is first determined in the tracered sample. The formula

$$N_{A}=\frac{N_{A^{\prime },T}}{\left(\frac{I_{A^{\prime },T}}{I_{A,T}}+\frac{I_{A^{\prime }}}{I_{A}}\right)}$$ 2

was used for this purpose. The number of atoms of the untracered isotope (^238^Pu, ^243^Am, ^244^Cm, and ^250^Cf), which is the same in the tracered sample and the untracered sample, is *N*_*A*_, and the number of atoms of the added tracer is *N*_*A*′,T_. The ratio $\frac{I_{A^{\prime },T}}{I_{A,T}}$ describes the isotope ratio of the tracered isotope *A*′ to the untracered isotope *A* in the sample to which the tracer was added, and $\frac{I_{A^{\prime }}}{I_{A}}$ describes the corresponding isotope ratio in the untracered sample. The isotopes observed as impurities in the tracers were not used for this analysis. The isotope pairs that were used to determine the isotope ratios in the tracered sample are shown in Figure 3b. In this manner, the absolute contents of ^238^Pu, ^243^Am, ^244^Cm, and ^250^Cf were determined. Based on these isotopes and the isotope ratios of the untracered sample as obtained from RIMS, ^239–242,244^Pu, ^241^Am, ^245–248^Cm, and ^249,251,252^Cf were then quantified. The quantities of these 17 isotopes in the total shipped sample are summarized in Table 3. The uncertainty of these values is on the order of 20% (1σ).

**Figure 3:**
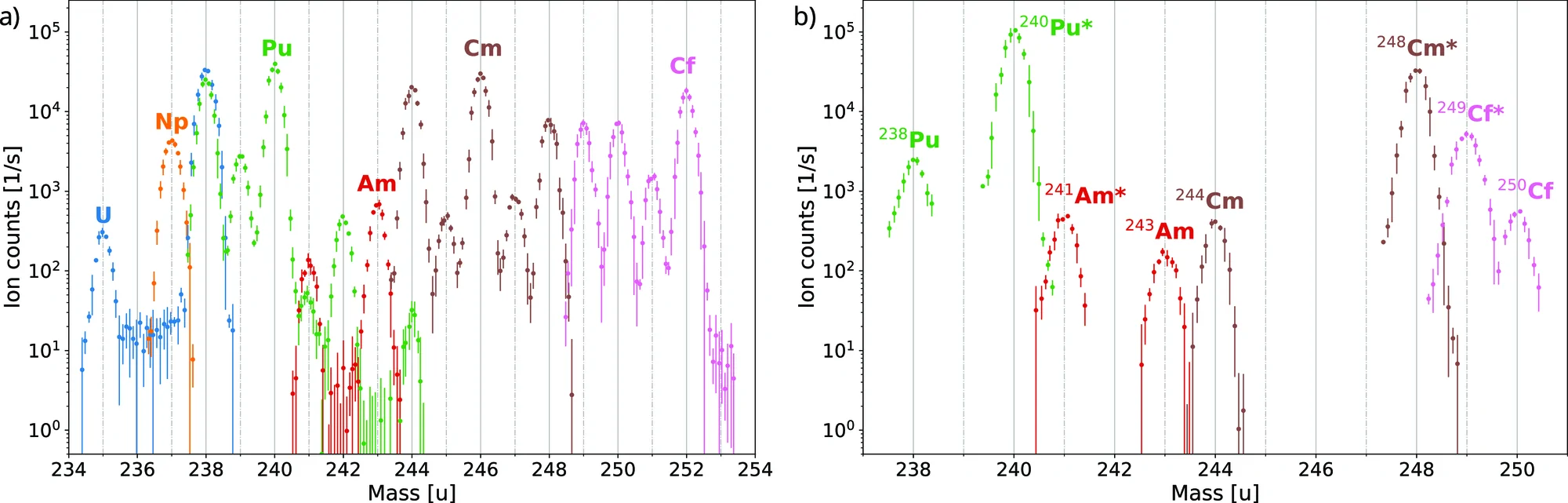
(a) Assembled mass scans of uranium, neptunium, plutonium, americium, curium, and californium measured via RIMS using 0.1% of the feed solution sample. (b) Compilation of mass scans of 0.1% of the feed solution sample with added ^249^Cf, ^248^Cm, ^241^Am, and ^240^Pu tracers. The tracered isotopes are marked with an asterisk, and the isotope of the corresponding element used for quantification is also shown.

The quantification of ^237^Np was somewhat more difficult, as no suitable tracer was available and as the ^241^Am tracer contains ^237^Np. Thus, a total efficiency measurement was performed for this quantification. To achieve this, another sample, containing 0.1% of the feed solution, was measured using RIMS. This time, the laser and magnet settings remained optimized for ^237^Np. The complete sample was evaporated and ionized and the count rate in the detector was registered and integrated over the whole evaporation time until the signal faded to background.

The ionization efficiency of the Np excitation scheme was determined in the same manner using samples quantified by α spectrometry. ^237^Np was then quantified by combining the integrally detected ^237^Np ions in the feed solution with the RISIKO efficiency for Np of 30(6)%, assuming that pure ^237^Np samples and the feed solution feature the same efficiency. The quantity of ^237^Np in the feed solution determined in this way is also given in Table 3. Due to possible matrix effects, the uncertainty was conservatively estimated to be a factor of two larger than the uncertainty of the efficiency for Np.

The application of RIMS allowed the absolute quantification of 14 isotopes that could not be measured with α or γ spectrometry, as shown in Table 3. The quantifications of the five isotopes ^238^Pu, ^244,246^Cm, and ^250,252^Cf, that were quantified by α spectrometry as well as by RIMS, were consistent within the uncertainty of the two methods. However, with regard to the quantification of ^238^Pu and ^244^Cm using α spectrometry, it should be noted that the α lines of ^238^Pu cannot be distinguished from those of ^241^Am, and the α lines of ^244^Cm cannot be distinguished from those of ^249^Cf. The atom numbers of ^241^Am and ^249^Cf were quantified using RIMS, which allowed correcting the amounts of ^238^Pu and ^244^Cm determined by α spectrometry. The corrections were negligible within the 10% uncertainty of the values; in the case of ^238^Pu, the amount determined by α spectrometry would be corrected by 4%, and in the case of ^244^Cm, the 2.7(3)×10^13^ atoms would be corrected by 0.9%.

In addition to the results from RIMS and α spectrometry, Table 3 shows the quantification of the feed solution from ORNL. Due to the time difference between the measurements at JGU and ORNL, the quantified values from ORNL were decay-corrected to the time of the RIMS measurements at Mainz. This is particularly necessary for ^252^Cf due to its short half-life of 2.647 a.^25^ For some isotopes, the quantification at ORNL resulted in slightly higher contents than those measured at JGU. However, the measurements of ^244^Cm and ^249,250,252^Cf are in agreement within 1σ, and those of ^238^Pu and ^251^Cf within 2σ. We thus conclude that the quantification at JGU using RIMS and α spectrometry is in good agreement with the quantification performed at ORNL by alpha spectrometry and ICP-MS, but extends the number of accessible isotopes significantly. Using RIMS, 19 isotopes of five elements were characterized and quantified. For 18 isotopes, this was done without extensive chemical separation of the sample, while for ^250^Cm, it was carried out after chemical separation of the Cm fraction from the sample.

## Summary

After intensive neutron irradiation of Cm targets in HFIR at ORNL, the feed solution was fully characterized and quantified by RIMS. For this analysis (except for ^250^Cm), 0.4% of the available sample was used. To quantify the low abundant ^250^Cm, a chemical separation of Cm form other actinides based on α-hydroxyisobutyric acid was performed, and 13% of the Cm fraction was used to quantify this isotope. The sample contained 5×10^7^ – 5×10^13^ atoms of the analyzed isotopes ^237^Np, ^238–242,244^Pu, ^241,243^Am, ^244–248,250^Cm, and ^249–252^Cf. With the classical radioanalytical methods, i.e., α and γ spectrometry, only six isotopes, ^238^Pu, ^244,246^Cm, and ^249,250,252^Cf were observed. Although the quantification of ^249^Cf was not possible. Overall, the results of the quantification using RIMS and the classical radioanalytical methods were consistent. Beyond that, and compared to other mass spectrometric methods such as ICP-MS, RIMS offers the advantage of lower sample consumption and does not require complex chemical separation for isobar suppression. However, quantifying ^237^Np using RIMS was inconvenient due to the lack of a suitable tracer. Possible Np isotopes that would be suitable as tracers are ^235^Np and ^236^Np, both of which must be produced in accelerator facilities.^26^ The β-decaying isotope ^249^Bk, which is essential for ^249^Cf production, was not identified in the sample neither using γ spectrometry nor RIMS. In the γ spectrum, the γ lines could not be identified due to the fission products of the spontaneous fission of ^252^Cf. In the case of RIMS, no suitable excitation scheme is yet known for berkelium. Thus, the quantification of ^249^Bk without chemical separation in such a complex sample is only possible via RIMS by developing suitable two-step excitation schemes for berkelium. Furthermore, the absolute ionization efficiency of these schemes must be determined, as no suitable tracer is available. Our results provide a benchmark for calculations carried out with the TCOMP and ORIGEN models, similar to what was reported in ref 27, where the amount of produced ^252^Cf was compared. Our analysis significantly broadens the range of actinides to be compared with these models, which forms a sound basis for optimized production of transcurium elements.

## Data Availability

The data underlying this study are openly available on Zenodo at https://doi.org/10.5281/zenodo.22698079.
